# Expression Loss and Revivification of RhoB Gene in Ovary Carcinoma Carcinogenesis and Development

**DOI:** 10.1371/journal.pone.0078417

**Published:** 2013-11-01

**Authors:** Yingwei Liu, Na Song, Kexing Ren, Shenglan Meng, Yao Xie, Qida Long, Xiancheng Chen, Xia Zhao

**Affiliations:** 1 Department of Gynecology & Obstetrics, West China Second Hospital, Sichuan University, Chengdu, Sichuan, People’s Republic of China; 2 National Key Laboratory of Biotherapy and Cancer Center, West China Hospital, Sichuan University, Chengdu, Sichuan, People’s Republic of China; Peking University Health Science Center, China

## Abstract

RhoB, a member of small GTPases belonging to the Ras protein superfamily, might have a suppressive activity in cancer progression. Here, expression of RhoB gene was evaluated in human benign, borderline and malignant ovary tumors by immunostaining, with normal ovary tissue as control. Malignant tumors were assessed according to Federation Internationale de Gynecologie Obstetrique (FIGO) guidelines and classified in stage I-IV. Revivification of RhoB gene was investigated by analyzing the effect of histone deacetylase (HDAC) inhibitor trichostatin (TSA) and methyltransferase inhibitor 5-azacytidine (5-Aza) on ovarian cancer cells via RT-PCR and western blot. Apoptosis of ovary cancer cells was detected using flowcytometry and fluorescence microscopy. Subsequently, RhoB expression is detected in normal ovary epithelium, borderline tumors, and decreases significantly or lost in the majority of ovarian cancer specimen (*P*<0.05). RhoB expression decreases significantly from stage II (71.4%) to stage III (43.5%) to stage IV (18.2%, *P*<0.05). TSA can both significantly revive the RhoB gene and mediate apoptosis of ovarian cancer cells, but 5-Aza couldn’t. Interference into Revivification of RhoB gene results in reduction of ovary carcinoma cell apoptosis. It is proposed that loss of RhoB expression occurs frequently in ovary carcinogenesis and progression and its expression could be regulated by histone deacetylation but not by promoter hypermethylation, which may serve as a prospective gene treatment target for the patients with ovarian malignancy not responding to standard therapies.

## Introduction

Ovarian cancer is the deadliest malignancy of the female reproductive tract and ranks fourth in causing female cancer mortality. Most of ovarian tumors originate from epithelium, although stromal and germ cell tumors also occur [1,2]. Ovarian cancer are generally asymptomatic in early-stage and diagnosis is usually (>80% of cases) established after the disease has disseminated out of the ovaries. The American Cancer Society's most recent estimates revealed 21,550 new cases of ovarian cancer and 14,600 deaths from the cancer in the United States in 2009. (www.cancer.org). Despite advances in surgery and chemotherapy, its five-year survival rates have changed little over the past decade, long-term survival is dramatically poor and remain among the worst of all anatomic site cancers. 

RhoB, a small guanosine triphosphatase (GTP)-binding protein, belongs to one of the subgroups of the Rho GTPase family, which regulates many cellular processes inclucding cytoskeletal organization, gene transcription, cytokinesis, and cell cycle progression[3,4]. Some recent studies confirmed the role of Rho proteins in cancer by showing their involvement in cell transformation, survival, metastasis, invasion and angiogenesis [5,6]. There are several features that distinguish RhoB from other Rho proteins. Although most Rho proteins have been shown to have positive role in proliferation and malignant transformation processes, RhoB rather appears to be a negative regulator [7,8]. RhoB is known to be immediate-early inducible by growth factors and protein-tyrosine kinases. RhoB displays some characteristics such as its localization to early endosomes [9], its rapid up-regulation by growth factors and genotoxic stress [10] and its role in intracellular transport of cell-surface receptors [11], which suggests that RhoB might have some special functions in transformed cells. RhoB mRNA and protein levels are turned over much more rapidly (half-lives of 20 and 120 min, respectively) than other GTPases, which have half-lives of about 24 h [12,13]. RhoB loss in mice was associated with an increased susceptibility to chemical carcinogenesis, and cells lacking RhoB were more efficient at forming intraperitoneal tumors [14]. The physiological functions of RhoB and other GTPases are predicted to be distinct. RhoB has been suggested as a potential gene suppressor candidate [15].

Loss of RhoB expression has been reported in other tumors such as Head and Neck carcinomas [16], brain tumors[17] and lung tumors [18]. The mechanism by which RhoB expression decreases in tumors is not elucidated so far. The first hypothesis to be investigated is that RhoB loss of expression is due to genetic mutation or deletion. However, only very few reports are available until now dealing with the analysis of Rho expression and Rho mutation in human tumours [19]. Previous study did not find any RhoB gene mutation in head and neck carcinoma [16], lung tumors [18] and breast tumors [20]. The other hypothesis is that RhoB expression is controlled by epigenetic mechanism. Wang et al. demonstrated that RhoB expression silence could be re-expressed by histone deacetylase inhibitor (HDACi) in lung cancer cell lines [21].

Based on the above mentioned, a hypothesis is coming into being that RhoB expression may be a marker to identify ovary cancer patients with high risk of developing metastases, as well as a prognostic marker useful in the clinic. In this study, human ovary tumor tissue ranging from different histological types of tumor to invasive tumors was analyzed for RhoB, with normal ovary epithelium as control, which showed that RhoB expression decreases with the progression of ovary cancer. In order to revive the expression loss of RhoB gene, in vitro ovarian tumor growth and treatment responses to HDACi and demethylating agent were observed. Interestingly, there was a significantly enhanced expression of RhoB after treated with HDACi, and meanwhile the growing tumor cells apparently decreased with obvious apoptosis. Thus, it is proposed that RhoB loss of expression be a frequent event in ovary cancer progression, which may serve as a useful target for gene therapy of ovarian malignancy.

## Results

### RhoB expression decreases or loss in ovary carcinoma

**Figure 1:**
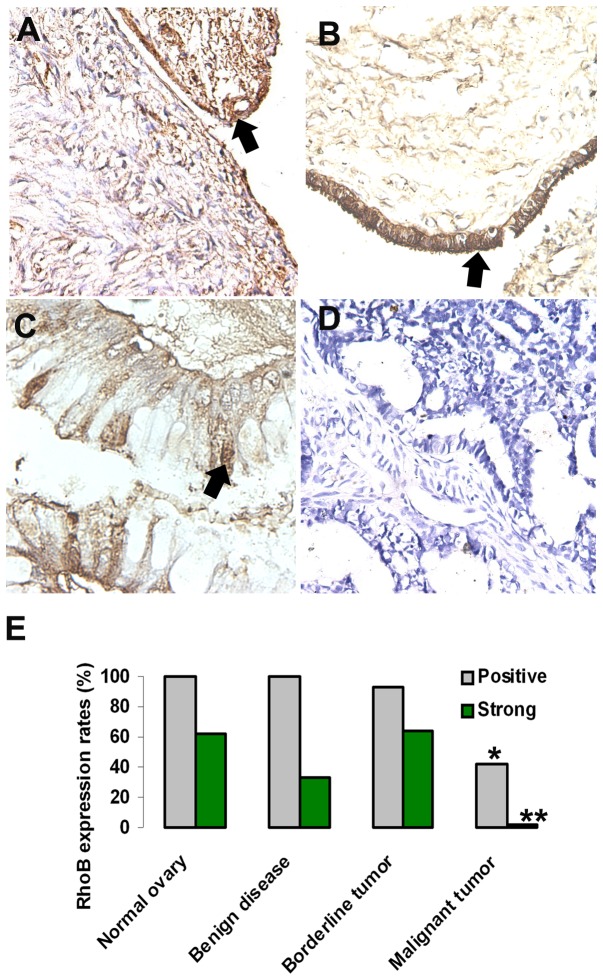
RhoB expression decreases or loss in ovary carcinoma development. RhoB expression levels were analyzed in normal ovaries, benign, borderline tumors and malignant ovary carcinoma respectively. Immunohistochemistry reveals that moderate or strong cytoplasmic expressions of RhoB were detected in most of the normal ovaries (A) and benign (B, serous cystadenoma) and borderline (C) tumors. Staining was well visualized along ovary epithelium (arrows), with negative (D) or weak expression in malignant invasive tumor. (E) It is clear that RhoB expression is detected in normal ovary epithelium (100%), benign diseases (100%), borderline tumors (92.8%), and decreases significantly in the malignant invasive tumors (42.1%, * *P*<05).

RhoB protein levels in 96 cases including normal ovaries, benign disease, borderline tumors and malignant ovary carcinoma were analyzed respectively to address the question of the relevance of RhoB in human ovary tumorigenesis (Table 1). Medium or strong cytoplasmic RhoB expression was detected in most of the normal ovaries, benign and borderline tumors. Staining was well visualized along ovary epithelium (Figure 1).

**Table 1:** RhoB protein levels in normal ovaries and tumor differentiotion.

**Tissues**	**Cases**	**Expression of RhoB %**				**Positive rate %**	**Strong positive rate %**
		<25	25-50	50-75	>75		
**Normal ovary tissue**	**16**	**0**	**2**	**4**	**10**	**100**	**62.5**
**Benign disease**	**9**	**0**	**1**	**5**	**3**	**100**	**33.3**
serous cystadenoma	6	0	1	4	1	100	16.7
mucinous cystadenoma	3	0	0	1	2	100	66.7
**Borderline tumors**	**14**	**1**	**1**	**3**	**9**	**92.8**	**64.3**
borderline serous cystadenoma	9	0	1	1	7	100	77.8
borderline mucinous cystadenoma	5	1	0	2	2	80.0	40.0
**Malignant tumors**	**57**	**33**	**15**	**8**	**1**	**42.1**	**1.75**
poor-differentiated	25	20	4	0	1	20	4
medium-differentiated	25	11	8	6	0	56	0
well-differentiated	7	2	3	2	0	71.5	0
serous carcinoma	33	20	9	4	0	39.4	0
mucinous carcinoma	19	11	5	3	0	42.1	0
Others	5	2	1	1	1	60	20

RhoB protein levels were detected in normal ovaries, benign disease, borderline tumors and malignant ovary carcinoma respectively.

**Figure 2:**
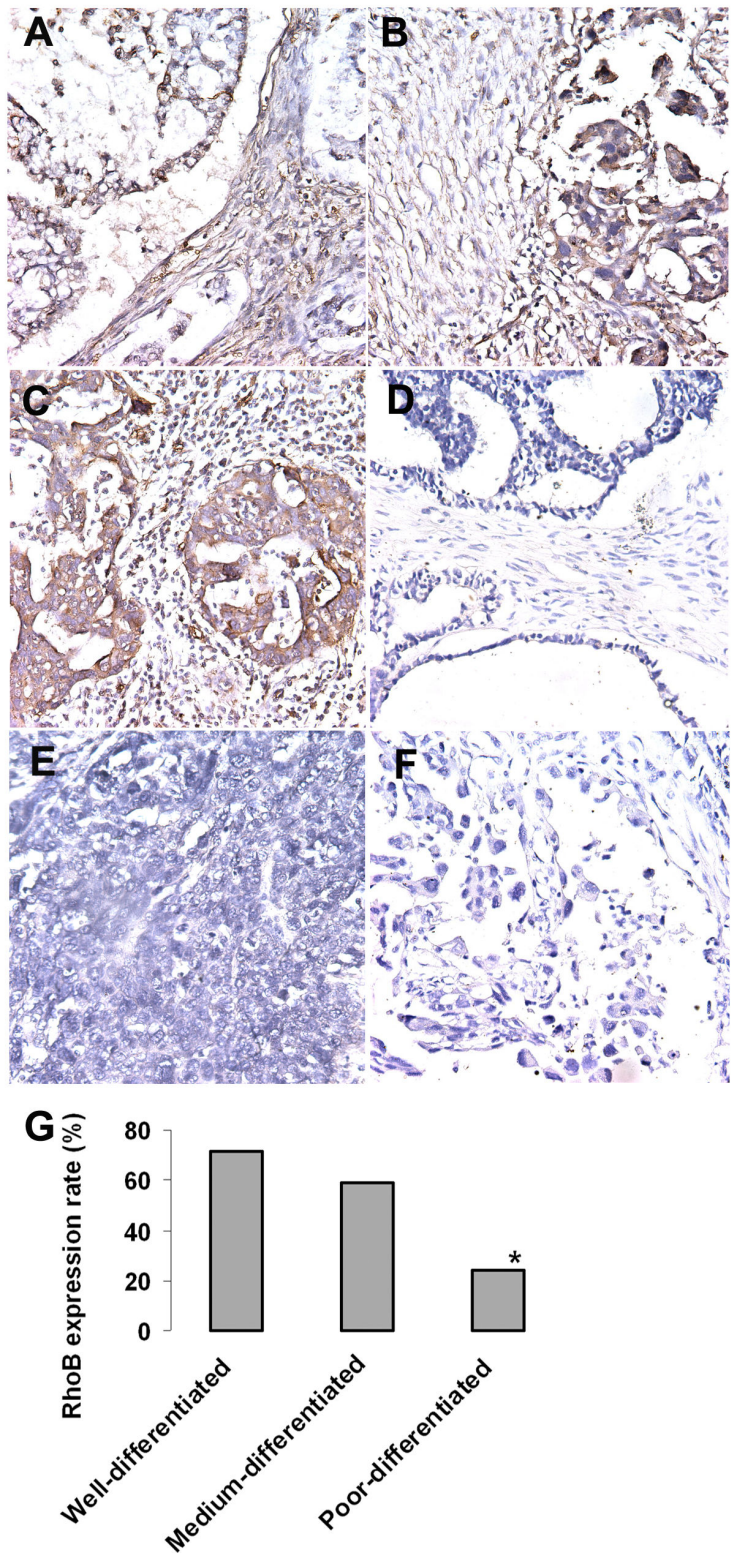
RhoB gene expression and ovary carcinoma differentiation. RhoB expression levels were analyzed respectively in well-, medium-, and poor-differentiated ovarian cancer using immunohistochemistry staining of individual paraffin sections. RhoB is expressed in the cytoplasm of monolayered ovarian surface epithelial cells for well-differentiated ovary caicinoma (AB); in medium-differentiated tumor, RhoB is expressed in the monolayered and multilayered regions of epithelium tumor (CD); and RhoB is lost in the multilayered region of epithelium for poor-differentiated tumor (EF). (G) It is clear that Poor-differentiated tumors had a significant reduction in the expression of RhoB (80% reduction) than medium differentiated (44% reduction) and well differentiated ovary cancer (28.5% reduction).

Out of 57 cases of malignant tumors, RhoB negative expression was detected in 27 cases, and only faint staining was observed in 14 cases. Obviously, the expression of RhoB was obviously reduced or lost in the majority of malignant tumors. No obvious difference was seen between various histological types. It is clear that RhoB expression is detected in normal ovary epithelium, borderline tumors, and decreases significantly in malignant tumors (*P*<05). Poor differentiated tumors had a greater (80%) reduction in the expression of RhoB than medium differentiated (44% reduction) and well differentiated ovary cancer (28.5% reduction). RhoB is expressed in the cytoplasm of monolayered ovarian surface epithelial cells, and its expression is lost in the multilayered region of epithelium (Figure 2). 

### Expression of RhoB and tumor stage

All of the tumors were assessed according to the FIGO and classified in stage I-IV. Stage I disease is rare because it is asymptomatic in early stage, thus after operation, 0 patients were clinically classified in Stage I, 7 in Stage II, 39 in Stage III, and 11 in Stage IV (Table 2). The expression of RhoB was determined in each stage, results from which indicate that RhoB expression decreases significantly (*P*<0.05) from stage II (71.4%) to stage III (43.5%) to stage IV (18.2%).

**Table 2:** Expression levels of RhoB and ovary carcinoma stage.

	**Cases**	**Expression of RhoB %**					**Positive rate %**	**Strong positive rate %**
		**<25**	**25-50**	**50-75**	**>75**			
**Stage I**	0	0	0	0	0		0	0
**Stage II**	7	2	4	1	0		71.4	0
**Stage III**	39	22	10	6	1		43.5*	2.5
**Stage IV**	11	9	1	1	0		18.2*	0

**Sum** 57 33 15 8 1

All of the tumors were assessed according to the FIGO and classified in stage I-IV. After operation, 0 patients were classified in Stage I since Stage I disease is rare in ovarian surgery because it is asymptomatic in early stage. 7 in Stage II, 39 in Stage III, and 11 in Stage IV. Stage I disease is rare because it is asymptomatic in early stage. RhoB expression decreases significantly from stage II (71.4%) to stage III (43.5%) to stage IV (18.2%, *P<0.05).

### HDACi revives RhoB gene and induces ovary carcinoma arrest

**Figure 3:**
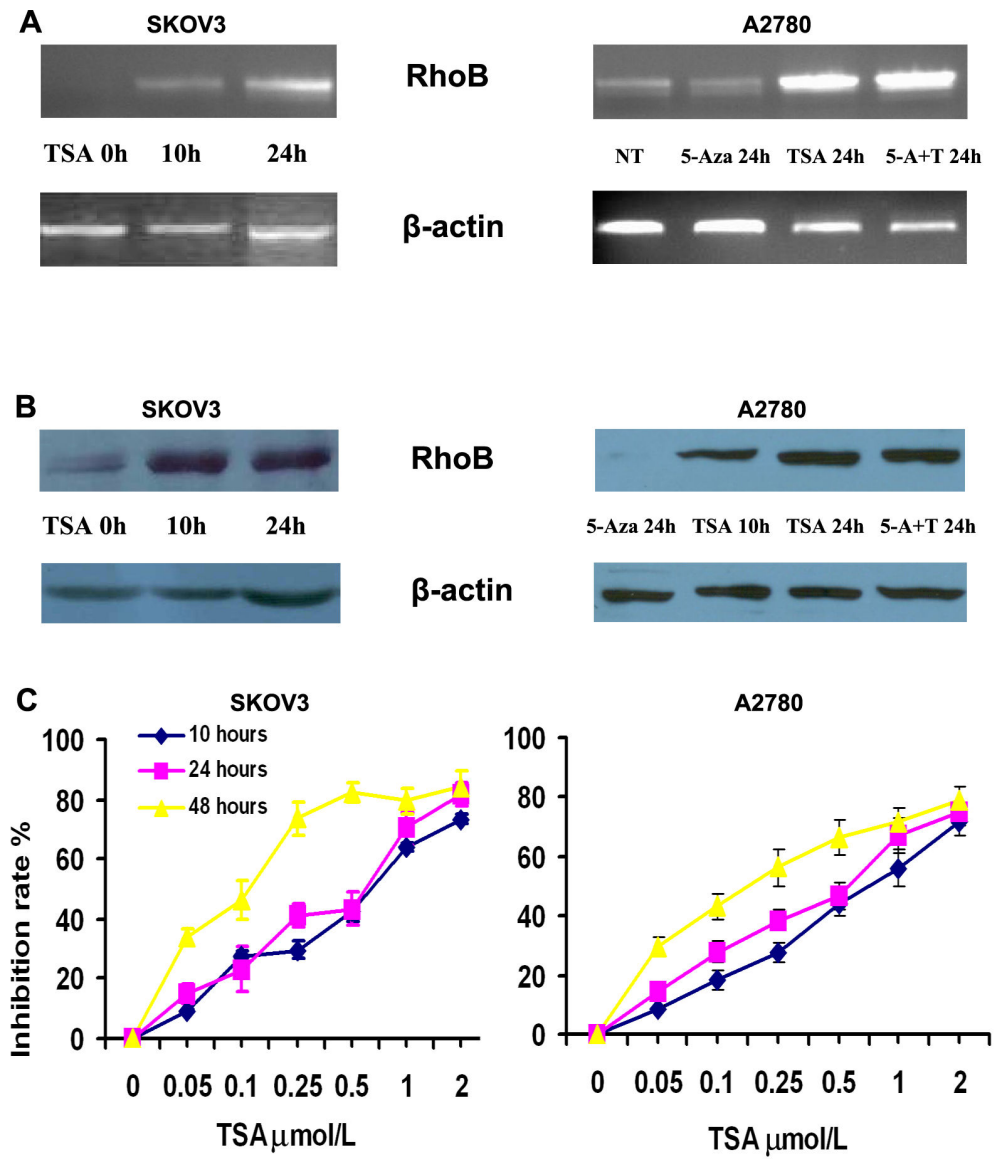
Restoration of expression loss of RhoB gene. Regulation of RhoB expression loss by histone deacetylase (HDAC) inhibitor trichostatin (TSA) and methyltransferase inhibitor 5-Azacytidine (5-Aza). (**A**) **Semi-quantitative RT-PCR analysis of RhoB expression levels in ovary carcinoma cell**: Cells were either nutreated (NT), treated with 5-Aza at 2 μM, with TSA at 0.5 μM, or with combination of TSA (0.5 μM) and 5-Aza (2 μM). It can be seen that 5-Aza did not increase RhoB expression. However, TSA could significantly reverse RhoB silencing in ovary cancer SKOV3 and A2780 cells. β-Actin was used as inner control. (**B**) **The expression confirmation of RhoB by Western blotting analysis**: SKOV3 and A2780 cells were exposed to 0.5μM TSA at different time points. β-actin was used as equal loading control. As shown, TSA treatment, but not 5-Aza, revived the protein expression of RhoB. (**C**) **TSA-mediated cell growth arrests of ovary cancer cells**: SKOV3 and A2780 cells were treated with gradient TSA for 10, 24 and 48 hours. The inhibition rate was calculated as a percentage of the *in* *vitro* proliferation in terms of untreated control cells by MTT. TSA inhibits SKOV3 and A2780 cells growth in a concentration- and time-dependent manner. The results were expressed as mean±S.D.

Since previous work has shown that RhoB loss of expression through ovary cancer progression and many tumor suppressor genes are silenced by epigenetic modification in ovary cancinogenesis [24], RhoB expression in ovary carcinoma SKOV3 and A2780 cell lines was analyzed after treatment with 5-Aza and TSA to see if the expression silence can be effectively revived by either promoter histone deacetylation or methylation. AS shown in Figure 3A-B, RT-PCR and Western-blot analyses indicated that treatment by 5-Aza could not revive RhoB gene expression. However, RhoB gene self-silencing in ovary cancer cells was significantly reversed by TSA. MTT assay demonstrated that TSA mediates growth arrest of SKOV3 cells in a concentration- and time-dependent manner (Figure 3C). 

### Interference into revivification of RhoB gene

**Figure 4:**
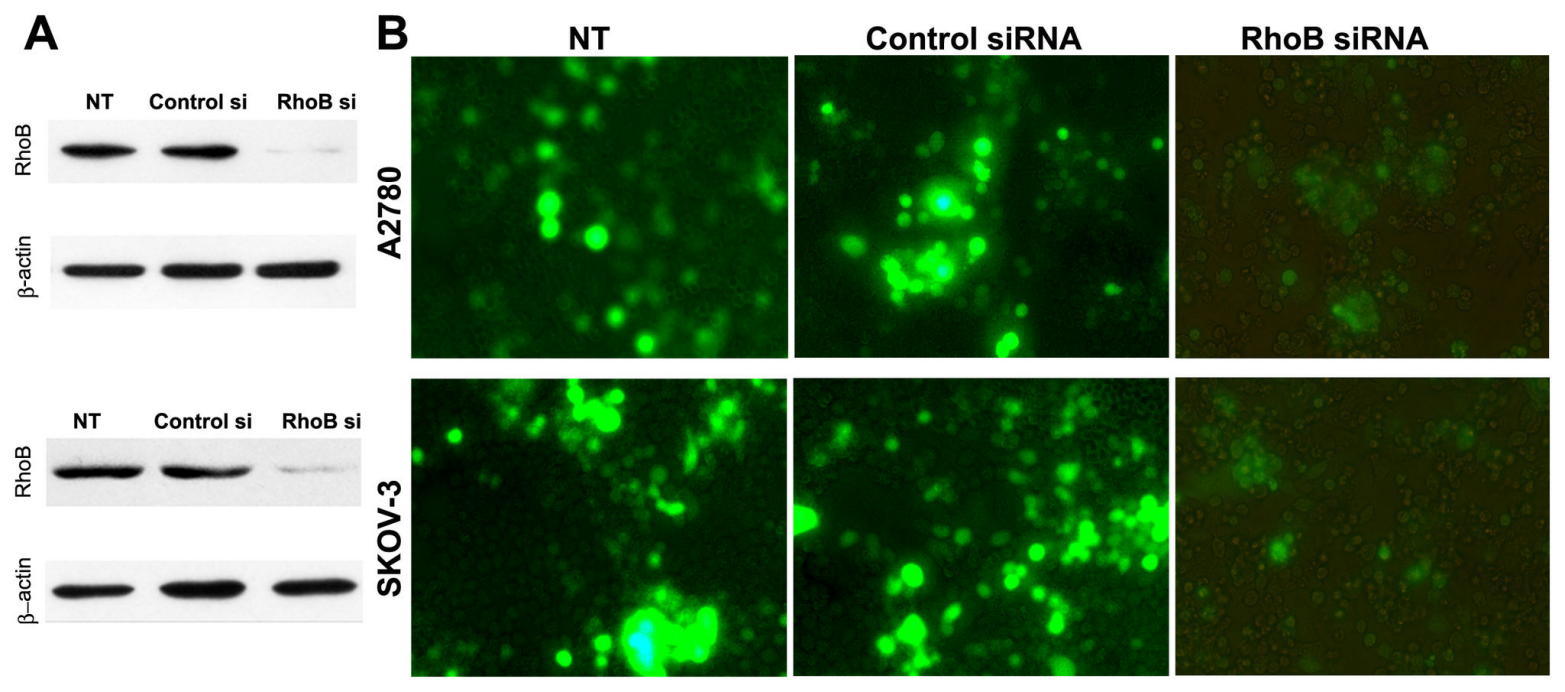
Interference into Revivification of RhoB gene results in inhibition of ovary carcinoma cell apoptosis. After treated with TSA for 24 hours, SKOV3 and A2780 cells were respectively either nu-transfected (NT), transfected with non-targeted Control siRNA (Control si), or with siRNA duplex against human RhoB (RhoB si). (**A**) **The expression confirmation of RhoB by Western blotting analysis with β-actin used as equal loading control**: As shown, transfection with RhoB siRNA, but not with Control siRNA, abrogate the protein expression of RhoB. (**B**) **Apoptosis assays of tumor cells after transfection with RhoB siRNA/Control siRNA following treatment with TSA**. TUNEL staining of carcinoma cells revealed that RhoB siRNA, yet not Control siRNA, caused a significant reduction of apoptotic cells in contrast to nu-transfected (NT) carcinoma cells.

Apoptotic assays after transfection with RhoB siRNA or Control siRNA next to treatment with TSA revealed that RhoB siRNA, yet not Control siRNA, lead to a significant reduction of apoptosis of revived-RhoB tumor cells in contrast to nu-transfected (NT) carcinoma cells (Figure 4). 

### HDACi induces ovarian tumor cell apoptosis

**Figure 5:**
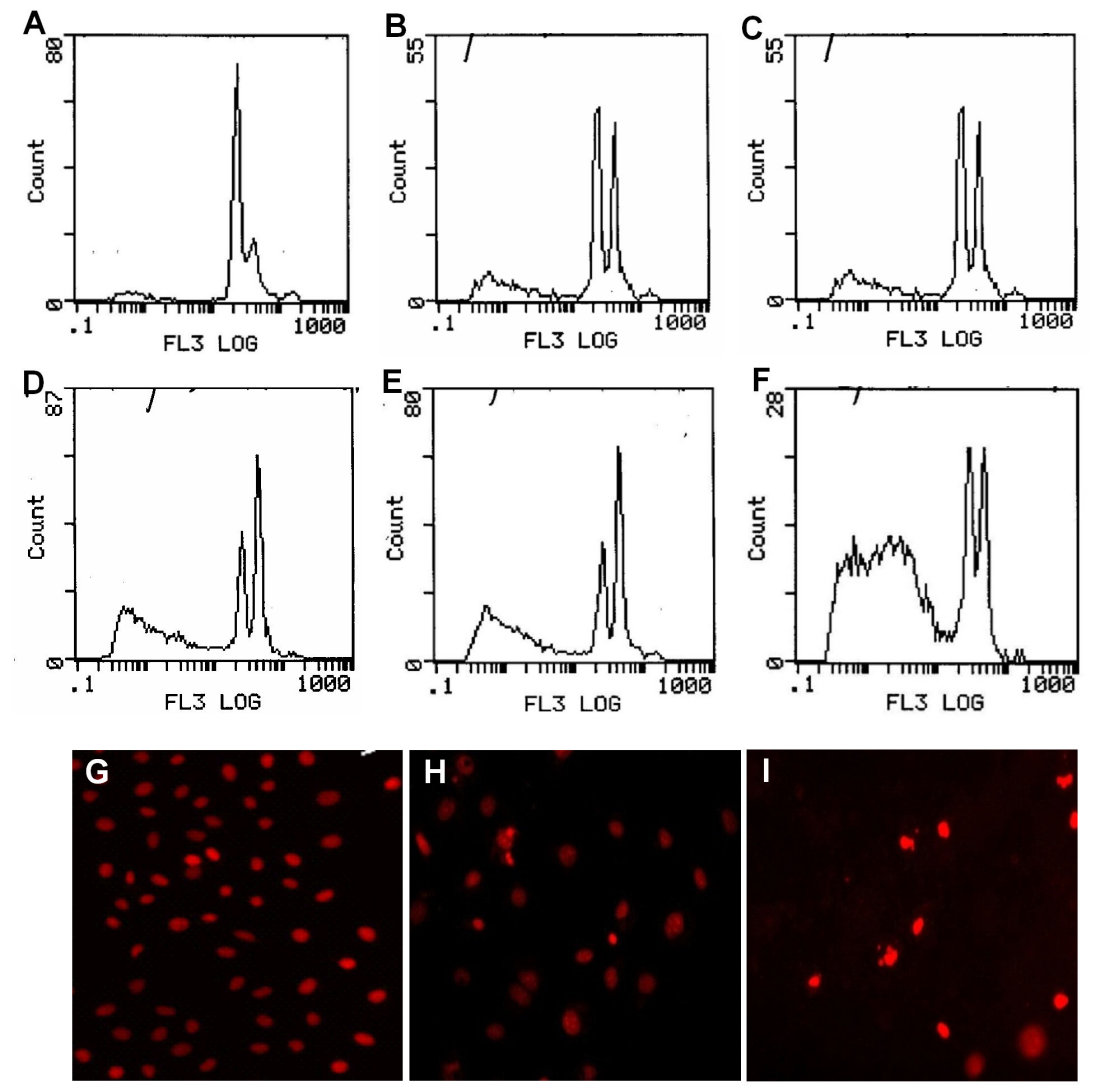
Revivification of RhoB gene and apoptosis of ovary carcinoma cells. Flow cytometric analysis (FCM) together with fluorescence microscopy was adopted to assess apoptosis of ovary carcinoma cells after treated with TSA. **Top and middle panels**: when cells were treated for 10 h with 0 µmol/L (control), 0.05 µmol/L, 0.1 µmol/L, 0.25 µmol/L, 0.5 µmol/L and 1.0 µmol/L of TSA, the apoptosis rate revealed by FCM was 9.5% (A), 26.9% (B), 28% (C), 41% (D), 45.9% (E) and 66.9% (F) respectively. **Bottom panel**: PI stained fluorescence photomicrographs of ovary carcinoma cells after treated with TSA. Cells were treated for 10h with 0 µmol/L (G, control), 0.1 µmol/L and 0.5 µmol/L of TSA respectively, both 0.1 µmol/L (H) and 0.5µmol/L (I) of TSA could result in cellular morphological changes characterized as apoptosis: a brightly red-fluorescent condensed nuclei (intact or fragmented), reduction of cell volume, and apoptotic bodies.

With the treatment by TSA for 10 h, the concentration leading to a 50% decrease in cell number (IC50) was about 0.5μmol/L. Based on the result, SKOV3 and A2780 cells were exposed to increase doses of TSA (0.05-1.0 µmol/L) for 10 h, and all the cells were harvested to measure apoptosis level by FCM. Even low dose of 0.05µmol/L TSA could cause 26.9 % apoptosis on the cells (p<0.05), and high dose (0.1, 0.25, 0.5 and 1.0µmol/L) of TSA could cause 28 %, 41%, 45.9% and 66.9% apoptosis respectively (Figure 5A-F). These data indicated that TSA could mediate apoptosis of ovarian tumor cells in a concentration-dependent manner. PI stained fluorescence photomicrography for the cells revealed morphological changes characterized as apoptosis after treated with TSA: a brightly red-fluorescent condensed nuclei (fragmented or intact), reduction of cell volume, or apoptotic bodies (Figure 5G-I). Results obtained in flow cytometry are strongly correlated with morphological changes in PI-stained fluorescence photomicrography.

## Discussion

As Rho-related members of the Ras-superfamily, RhoA, RhoB, and RhoC share high sequence identity. These RhoGTPases have been involved in the progression of tumors from a broad range of tissue origins, and analyses at both the mRNA and protein level have correlated their changed expression with tumor progression [25]. As positive regulators in proliferation and malignant transformation processes, most Rho proteins are involved in promoting oncogenesis, invasion and metastasis, but accumulating evidence points to a tumor-suppressive role for RhoB. By contrast to RhoA and RhoC reported to be up-regulated in several types of cancers, RhoB displays a property that may participate in tumor suppression [26]. Silencing of RhoA induced a strong up-regulation of both total and active RhoB protein levels that could be reversed by re-expressing RhoA and related to an enhanced half-life of the protein [27,28]. The RhoA-dependent regulation of RhoB does not depend on the activity of RhoA but is mediated by its GDP-bound form, so this regulation is only visible upon the depletion of inactive pool of RhoA. RhoB concentration was also slightly increased upon RhoC silencing, whereas the double silencing of RhoA and RhoC induced a dramatic increase of RhoB as compared with the single silencing of RhoA. RhoB alterations of post-translational prenylation by inhibitors of the mevalonate pathways or farnesyltransferase can enhance the expression of RhoB by acting at transcriptional level, but also through modulation of the protein stability [29].

In patients with head and neck, lung, or brain cancers, RhoB protein levels are decreased as the tumors become more aggressive and invasive [16,17]. RhoB-/- mice have increased susceptibility to Ras-induced tumorigenesis [14], while overexpression of RhoB suppresses Ras-mediated transformation [15,30]. Loss of RhoB is often found in human cancers during tumor progression. Wheeler AP et al. reported that deletion of RhoB affects the cell adhesion, spreading and migration speed, and that these effects are dependent on the substrate availability, correlating with reduced surface integrin levels [31]. DeWard AD et al. show that the additional knockout of RhoB expression in Drf1-null mice accelerates the progression to myelodysplasia (MDS) [32]. The small GTPase RhoB is required for apoptosis in response to DNA damaging agents and farnesyltransferase inhibitors [14,33].

The vast majority of ovary cancers (85%) arise from specialized epithelial cells that cover the surface of the ovary [34]. Many of the genes are abnormal expressed or silenced in cancer cells due to the epigenetic modifications of the genome [35]. Couderc et al. reported that in vitro reintroduction through recombinant adenovirus transduction could result in the apoptosis of endogenous RhoB low-expressing ovarian adenocarcinoma cells (OVCAR-3/IGROV-1) through the intrinsic apoptotic caspase cascade activation [36]. 

Histone acetylation induced by histone acetyltransferases (HATs) is associated with the gene transcription, while histone hypoacetylation induced by histone deacetylase (HDAC) activity is associated with gene silencing. The presence of acetylated lysine in histone tails is associated with relaxed chromatin state and gene-transcription activation, while the deacetylation of lysine residues is associated with a more condensed chromatin state and transcriptional gene silencing. Several specific HDAC inhibitors (HDACi) such as trichostatin A (TSA) has been shown to modify reversibly or irreversibly the balance between HAT and HDAC activities [37], and may dramatically regulate cell growth and induce differentiation and apoptosis, which is currently evaluated in clinical trials. In the past years, some studies have shown that TSA could induce growth arrest, differentiation, and apoptosis in a lot of cancer cells, including lung [38], colon [39], breast [40], and leukemic cells [41]. 

Recent studies show that RhoB is critical for promotion of stress-induced apoptosis and antineoplastic activity, and that loss of RhoB compromises the response of embryonic fibroblasts to stress stimuli [42]. This suggests that RhoB may act as a negative moderator of cell survival [7]. RhoA-related regulation of RhoB could be operational in cells exposed to bacterial toxins such as *Clostridium difficile* toxin B (TcdB). The cells treated with 1 ng/ml of TcdB, the RhoA level decreases with a parallel increase in the RhoB level. Huelsenbeck et al. demonstrated that the apoptotic effect of TcdB was mediated by RhoB [43]. 

In our study, Epigenetic regulation of RhoB expression was investigated by analyzing the effect of histone deacetylase (HDAC) inhibitor trichostatin (TSA) and methyltransferase inhibitor 5-Azacytidine (5-Aza) on ovarian cancer SKOV3 and A2780 cells via RT-PCR and western blot. RhoB was strong expressed in normal ovary tissue, whereas in ovarian cancer the RhoB was significantly decreased with ovary carcinogenesis. More importantly, TSA could significantly increase the expression of RhoB and decresed cell proliferation of ovarian cancer cells. As for the effects of TSA and 5-Aza on the morphology and proliferation of SKOV3 and A2780 ovarian cancer cells, our data indicate that TSA, but not 5-Aza, could induce a significant change in cellular morphology and inhibition of cell cycle progression. Apoptosis of ovary cancer cells was detected using flow cytometry and fluorescence microscopy. Our data indicated that TSA could mediate apoptosis of ovarian tumor cells in a concentration-dependent manner. It is proposed that loss of RhoB expression occurs frequently in ovary carcinogenesis and progression and its expression is mainly regulated by histone deacetylation rather than by promoter hypermethylation. 

Above all, efficacy of TSA on ovarian tumor cells indicates that epigenetic modifications that result from hypoacetylation of histone proteins may play a critical role in the formation of ovarian cancers. In our study, RhoB was hardly expressed or lost in clinic ovary cancer, whereas RhoB gene was evidently up-regulated in SKOV3 and A2780 cells after treated with TSA. Additional researches are being conducted to evaluate the RhoB function in ovarian cancer progression and the potential use of this gene as a selectable target that could provide gene treatment options for patients with ovarian tumors.

## Materials and Methods

### Sample collections

The study consisted of a total of 96 cases obtained from the Pathology Department at the West China Second Hospital of Sichuan University from the year 2005 to 2008. It was approved by the institution’s Institutional Review Board (IRB) of the West China Second Hospital of Sichuan University. The informed consents were written down and all the documents were kept in West China Second Hospital. Tissue sections were placed by the study pathologist in the following categories, 16 normal ovary tissues, 9 benign diseases (6 serous cystadenoma, 3 mucinous cystadenoma), 14 borderline tumors (9 borderline serous cystadenoma, 5 borderline mucinous cystadenoma), 57 malignant tumors (33 serous carcinoma, 19 mucinous cacinoma, 5 others). These primary malignant tumors were divided into three histologic grades of malignancy according to the degree of differentiation of tumor tissues, 7 well differentiated, 25 medium differentiated, 25 poor differentiated. All of the specimens were obtained from surgical resection. Histological diagnosis was assessed by an ovary cancer pathologist according to the last WHO classification [22], with all the patients assigned for tumor clinical stage via applying the Federation Internationale de Gynecologie Obstetrique (FIGO) guidelines [23] and classified in stage I-IV. All patients did not receive any neo-adjuvant treatment (neither chemotherapy nor radiotherapy) before surgical resection and followed-up after tumor resection to ensure collection of clinical data. 

### Immunohistochemistry

Immunohistochemistry was performed on formalinfixed tissues. Sections (5μm) of tissue microarray blocks were deparaffinized in xylenes and rehydrated in serials dilutions of ethanol. Endogenous peroxidase activity was blocked by incubation in 3% H_2_O_2_ for 10 minutes at room temperature. Antigenic sites were revealed after the tissue microarray sections were heated in a microwave processor at 97 °C twice for 5 minutes in citrate buffer solution. The primary monoclonal mouse anti-human RhoB (diluted 1:200, C5, Santa Cruz Biotechnology) was applied for 2 hours at 37 °C after the sections have been incubated with rabbit nonimmune serum for 10 minutes at room temperature. The sections were then incubated with avidin-biotin peroxidase complex for 30 minutes at 37 °C after incubated with the secondary biotinylated rabbit anti-mouse antibody (diluted 1:200) for 40 minutes at 37 °C. Then 3, 3-diaminobenzidine (DAB) was used as the chromogenic substrate then. The sections were counterstained with hematoxylin, dehydrated with serials dilutions of ethanol and put under a covership. Negative controls were processed with PBS instead of the primary antibody.

Immunostaining was evaluated by a semiquantitative method according to the percentage of positive tumors cells, the intensity was classified as follows: lack of staining (<25% -), mild staining (25-50% +), moderate staining (50-75% ++), and strong staining (>75% +++). Results were assessed and confirmed by two separate experienced pathologists. This scoring system has been validated previously (16/18). A diffuse cytoplasmic staining was scored as positive reactivity for RhoB.

### Cell lines

Human epithelial ovarian cancer cell lines SKOV3 and A2780 were respectively purchased from the American Type Culture Collection (ATCC, Manassas, VA) and European Collection of Cell Cultures (ECACC; Salisbury, UK) and both preserved by the State Key Laboratory of Biotherapy. Cells were cultured in a humidified atmosphere containing 5% CO_2_ at 37°C in DMEM supplemented with 10% FBS, 2mM L-glutamine, 100 units/ml of penicillin, and 100 μg/ml of streptomycin. Cultures were periodically tested to ensure they remained free of mycoplasm infection during the course of the experiments. 

### Reviving assay of RhoB gene in ovary cancer cells

In order to revive the expression loss of RhoB gene in ovary cancer cells, treatments with trichostatin A (TSA) and 5-Azacytidine (5-Aza) were adopted. TSA and 5-Aza, supplied by Sigma (St. Louis, MO), were dissolved in DMSO respectively, with final concentration of DMSO below 0.1%. 5-Aza treatment was performed as following, briefly, cells were treated for 24 hours with 2μM of 5-Aza, or treated with 0.5μM of TSA for 10, 24, 48 hours before analysis. TSA plus 5-Aza combination experiments were done by treating cells with 5-Aza, and TSA was added in the last 10 hours. 

### The silencing of Revived RhoB gene using siRNA transfection

SKOV3 and A2780 cells were respectively transfected with lyophilized siRNA (h) duplex against human RhoB (Sc-29472, Santa Cruz) resuspended in RNAse-free water according to siRNA Transfection Protocol (Santa Cruz Biotechnology, Inc.) or with non-targeted Control siRNA-A (Sc-37007, Santa Cruz) following the treatment with TSA for 24 hours. Apoptosis was evaluated by the TUNEL (terminal deoxynucleotidyl transferase-mediated dUTP nick endlabeling) technique using the DeadEnd Fluorometric TUNEL System (Promega, Madison, WI) according to their manufacturer’s protocol.

### Reverse transcription PCR

Total RNA from ovary cancer cell lines SKOV3 and A2780 was extracted using Trizol reagent (invitrogen, USA). According to manufacturer’s protocol, the total RNA was precipitated, then washed and dissolved in RNase-free water. The RNA solution was immediately subjected to RT-PCR amplification in GeneAmp PCR system 9700 with Two-step PCR kit from Progama and performed using the conditions as follows: reverse transcription at 48 °C for 30 min, and boiling at 94 °C for 2 min; then amplification for 30 cycles at 94 °C for 0.5 min, annealing at 59 °C for 0.5 min, and extension at 72 °C for 0.5 min; then completing the elongation at 72 °C for 10 min and finally held at 4 °C. Primers for reverse transcription-PCR were obtained from invetrogen, Inc. (Alameda, CA). Primer sequences for the human RhoB cDNA were 5'-TCGTAAGCCCAATTAAGGGGT-3' (forward) and 5'-GCTCTCTCCCGGGTCTCTCCG-3' (reverse). The house-keeping gene β-actin was tested as the inner control.

### Western-blot

Cells treated with TSA and 5-Aza were washed in cold PBS twice and harvested in cell lysis buffer (50 mmol/L Tris-HCl pH 7.4, 1% Triton x-100, 150 mmol/L NaCl, 1% deoxycholate plus 25 ug/mL leupeptin, 2 mmol/L EDTA, 10 ug/mL aprotinin, and 1 mmol/L sodium orthovanadate). Cellular protein was quantitated by Bradford assay (Biorad), and 50 μg of the cleared lysates were separated on a 12% SDS-PAGE, and electro-transferred onto PVDF membranes (Amersham Pharmacia Biotech). PVDF (Millipore, Bedford, MA) membranes were blocked in Tris-buffered saline containing 0.1% Tween 20 (TBST) with 5% nonfat dry milk for 2 hours, and incubated with monoclonal antibodies against RhoB (C5, Santa Cruz Biotechnology). Then the membranes were washed for 5 min in PBST for 3 times, followed by incubation with goat anti-mouse/rabbit secondary antibody at 1:5000 dilution (abcam) in PBST for 1 h. After washing 3 times with PBST and 2 times with PBS for 5 mins, the bands were developed using the enhanced chemiluminescence (ECL) detection system (Pierce Biotech Inc., Rockford, IL) according to the manufacturer’s instruction.

### MTT assay and apoptotic assessment in vitro

The MTT assay was done to investigate the resistance of SKOV3 and A2780 cells. For TSA, IC50 (24h) of the cells was 0.5μM. Cells (5×10^4^) were grown in 96-well plates and incubated for 24 h to about 70% confluence, then the cells were incubated with gradient TSA (0µmol/L, 0.05µmol/L, 0.1µmol/L, 0.25µmol/L, 0.5µmol/L, 1.0µmol/L, 2.0µmol/L) for 10, 24, 48 hours. The inhibition rate revealed with MTT assay was calculated as a percentage of the proliferation in terms of untreated cells as control. Flow cytometry (FCM) was used together with fluorescence microscopy to detect the apoptotic rate of tumor cells after treated by TSA. Quantitative evaluation of cellular apoptosis was performed by FCM using propidium iodide (PI)-staining method. After the treatment, cells were harvested, washed, suspended in cold PBS. Then cell samples were stained with 5 mg/ml PI and analyzed on a FACScan flow cytometry system (Becton Dickinson, San Jose, CA, U.S.A.). Data were analyzed using winMDIv2.8 software. Results were tested for statistical significance by Dunnett-t test. Significance was defined as P<0.05. The morphological changes of TSA-treated cells were observed by PI-stained fluorescent microscopy. Image of cells was taken by using ZEISS AXIOVERT 200 microscope and Axio Cam MRm camera.
